# Hematologic Profiles of Ethiopian Preterm Infants With Clinical Diagnoses of Early-Onset Sepsis, Perinatal Asphyxia, and Respiratory Distress Syndrome

**DOI:** 10.1177/2333794X20960264

**Published:** 2020-09-28

**Authors:** Zemene Tigabu Kebede, Yohannes Hailu Matebe, Abayneh Girma Demisse, Mulugeta Ayalew Yimer, Amha Mekasha, Alemayehu Worku, Asrat Demtse Gebremedhin, Elizabeth M. McClure, Assaye K. Nigussie, Bogale Worku, Netsanet Workneh Gidi, Gesit Metaferia, Robert L. Goldenberg, Lulu M. Muhe

**Affiliations:** 1 University of Gondar, Gondar, Ethiopia; 2 Addis Ababa University, Addis Ababa, Ethiopia; 3 RTI International, Durham, NC, USA; 4 Bill and Melinda Gates Foundation, Seattle, WA, USA; 5 Ethiopian Pediatric Society, Addis Ababa, Ethiopia; 6 St Paul’s Hospital Millennium Medical College, Addis Ababa, Ethiopia; 7 Jimma University, Jimma, Ethiopia; 8 Columbia University, New York, NY, USA

**Keywords:** preterm, complete blood count, clinical diagnosis, low-resource countries

## Abstract

*Objective.* To determine the hematologic profile of preterm infants with regard to different diseases. *Methods.* A prospective, cross-sectional, observational study, conducted in 5 hospitals in Ethiopia from July 2016 to May 2018. Preterm babies <7 days of age were included and investigated with complete blood counts (CBC) and other investigations, accordingly. *Results.* Out of 4919 preterms, 3852 (78.3%) were admitted to a newborn intensive care unit, and of these, 68.3% had a CBC performed. The mean values of hemoglobin, white blood cell (WBC) and platelet counts were 17.9 mg/dL; 12 685 cells/mm^3^, and 159 340 cells/mm^3^, respectively. Early onset neonatal sepsis (EONS) 1433 (37%), asphyxia 266 (6.9%), and respiratory distress syndrome (RDS) 1738 (45.3%) were common reasons for admission. The WBC count was <5000 cells/mm^3^ for 8.8%, 9.0%, and 11.1% of neonates with EONS, asphyxia and RDS, respectively. The hemoglobin value was <7 mg/dL for 0.6%, 1.7%, and 0.4% of preterm infants with EONS, asphyxia, and RDS, respectively. The platelet count was <50 000 cells/mm^3^ for 16.8%, 17.7%, and 19.8% of preterms admitted with a diagnosis of EONS, asphyxia, and RDS, respectively. *Conclusion*. WBC and platelet counts were the most common to be associated with EONS, asphyxia, and RDS. Further study is recommended to determine the effect of abnormal hematologic profile on the outcome of preterm babies.

## Introduction

Various hematological parameters such as the hemoglobin (HGB) level, white blood cell (WBC) count, and platelet count are diagnostic tools commonly used to evaluate preterm neonates. These tests are easily available as rapid screening tests and are reported to have good sensitivity and negative predictive values.^1,2^

Hematologic profiles of neonates can be affected by many factors such as complications of prematurity, infection, postnatal fluid shifts, late clamping of the umbilical cord, sampling sites, type of delivery, and the timing of the sample collection. The profile can also be affected by conditions such as hypothermia, asphyxia, and other prematurity related problems.^3^

Maternal complications such as preeclampsia and intrauterine growth restriction accompanied by chronic hypoxia may stimulate the generation of reticulocytes and reduce the number and total mass of megakaryocytes, as well as blunt the function of platelets.^4,5^ Infection and inflammation may increase the number of platelets and then increase their consumption.

The variation in the value of each of the hematologic profiles likely has an impact on the outcome of preterm babies. However, the hematologic profile of preterm neonates is not well-known and thus, the normal values of term babies are often used as the standards.

Prematurity is the single most significant factor correlated with neonatal sepsis. Neutropenia may be a significant finding with an ominous prognosis when associated with sepsis. Thrombocytopenia is usually a late and nonspecific sign of sepsis. Inflammation due to many reasons affects erythropoiesis by impairing iron metabolism. Anemia and sepsis are potential triggers for apnea of prematurity.^6^

Hematological parameters are the first to be affected in perinatal asphyxia, often within the first hour of delivery, even before other systemic changes occur. They are the most simple and feasible parameters to be evaluated. Among the hematological parameters, nucleated red blood cells (RBCs) and the total WBC count are found to be good prognostic indicators of perinatal asphyxia.^7^

Neonates with respiratory distress syndrome commonly have low oxygenation levels because of insufficient (or dysfunctional) surfactant, where they develop generalized atelectasis, ventilation-perfusion mismatching, with subsequent hypoxemia and respiratory acidosis.^8^ They also may have a HGB significantly different from normal.

The variation in the hematologic profile of preterm babies with regard to the common complications of prematurity is not well known. Therefore, the aim of this study is to determine the hematologic profile of preterm neonates with respect to sepsis, asphyxia and respiratory distress syndrome.

## Materials and Methods

The study was conducted in 5 different hospitals in Ethiopia, including Tikur Anbessa Hospital, Ghandi Memorial hospital and St. Paul Hospital Millennium Medical College, Jimma University Medical Centre, and Gondar University Hospital.

This prospective, observational study is a secondary evaluation of the SIP study.^9^ The study included all preterm babies delivered in the study hospitals, and others who were referred to one of the study hospitals before 7 days of life from July 2016 to May 2018. Participants were excluded if delivery was a result of an induced abortion or the gestational age could not be reliably established using the study criteria. Details of the protocol for the SIP study have been published elsewhere.^9^

For the analysis, we limited the population to the infants who underwent laboratory investigations of a complete blood count (CBC) using an automated SYMEX machine.

A 2 mL blood sample was taken in an EDTA vacutainer and was processed for HGB, WBC, and platelet count with an automated machine. The normal WBC count for newborns is reported to range from 9000 to 30 000 cells/mm^3^ at the time of birth and for this study, leukopenia refers to a WBC count <5000 cells/mm.^3,10^ At birth, normal values for the central venous HGB in infants of >34 weeks’ gestational age are 14 to 20 g/dL, with an average value of 17 g/dL.^11^

The platelet count varies based on gestational age. The mean platelet count is reported to be ≥200 × 10^3^/μL in most preterm infants, and the 5th percentile is found to be 104 × 10^3^/μL for those ≤32 weeks’ gestation, and 123 × 10^3^/μL for late preterm and term neonates.^12^ Neonatal thrombocytopenia is defined as a platelet count of <150 × 10^3^/μL and is classified as mild (100-149 × 10^3^/μL), moderate (50-99 × 10^3^/μL), or severe (<50 × 10^3^/μL). Other investigations were done as clinically indicated.^13^

### Operational Definitions

Perinatal asphyxia: A neonate having an acute intrapartum event sufficient to cause neonatal injury evidenced by a need for resuscitation, prolonged depression after birth (APGAR score <7 at 5 minutes) and with evidence of neonatal encephalopathy.^14^

Early onset sepsis: A constellation of signs and symptoms in the first 7 days of life making up the systemic inflammatory response syndrome in the presence of or as a result of suspected or proven infection.^15^

Respiratory distress syndrome: A neonate of less than 37 weeks of gestation presenting with tachypnea of ≥60 breaths per minute, tachycardia, increased breathing effort, bilaterally decreased air entry and/or cyanosis shortly after birth with diffuse, fine reticulogranular (ground-glass) densities, low lung volumes and air bronchograms on chest x-ray when available.

Leukocytosis WBC* count > 25 000 cells/mm^3^
Leukopenia WBC count < 5000 cells/mm^3^
Polycythemia  HGB*> 22 mg/dL
Anemia HGB < 15 mg/dL
Thrombocytosis Platelet count of > 450 × 10^3^/μL
Thrombocytopenia Platelet count of <150 × 10^3^/μL
*WBC = white blood count, *HGB = hemoglobin

## Data Analysis

At each hospital, data were double entered into study computers using the data management system developed for this study. Data were transferred on a regular basis from each hospital’s data management computer to the data center at Addis Ababa University, creating a complete data repository. Data were merged into one master data set for analysis. Data were analyzed using EXCEL software for determination of frequencies and proportions in regard to clinical diagnosis for calculation of the hematologic profile.

## Ethical Considerations

All clinical procedures were conducted per hospital protocol. The study was approved by the Institutional Review Boards of each hospital and the College of Health Sciences of the Addis Ababa University (MF03-008). Informed and written consent was obtained from parents or caretakers prior to the infants’ participation in the study. Consent information was available in English, Amharic or Oromifa languages, as appropriate. Confidentiality of the information was maintained.

The study was supported by a grant from the Bill & Melinda Gates foundation

## Results

**Figure 1.**
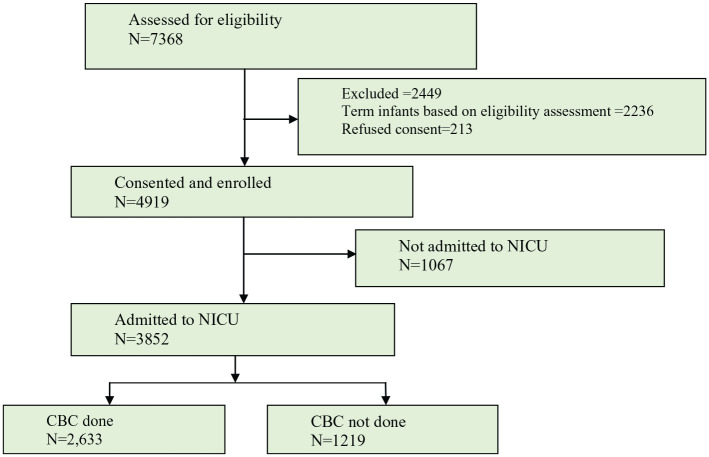
Project flow diagram.

A total of 4919 preterm infants were recruited into the study from July, 2016 to May, 2018. Of these, 3852 were admitted to one of the study NICUs. Of the total admissions, 2633 (68.35%) had a CBC determined and were included (Figure 1). The mean values for, HGB, WBC, and platelets were17.9 mg/dL, 12 747 cells/mm^3^ and 159 340 cells/mm^3^, respectively.

In this study, the top 3 reasons for admission were early onset neonatal sepsis (EONS) 1433 (37.0%); perinatal asphyxia (PNA) 266 (6.9%) and respiratory distress syndrome (RDS) 1738 (45.3%) (Table 1). Out of 1738 admitted neonates with a diagnosis of RDS, the WBC count was determined for 75.6%, and of 1433 neonates admitted with a diagnosis of EONS, a WBC was determined for 87.3%, and of 180 neonates with a diagnosis of PNA, a WBC was determined for 67.7%.

**Table 1.** NICU Admission Diagnoses With CBC Performed.

Admission Diagnosis	CBC Done	CBC Not done	Total
EONS	1251 (87.3%)	182	1433
PNA	180 (67.7%)	86	266
RDS	1314 (75.6%)	424	1738

Abbreviations: CBC = complete blood count; EONS = early onset newborn sepsis; PNA = perinatal asphyxia; RDS = respiratory distress syndrome.

The WBC count was <5000 cells/mm^3^ for 109 (8.8%), 16 (9%), and 144 (11.1%) of neonates diagnosed with EONS, PNA, and RDS, respectively. The WBC count was >25 000 cell/mm^3^ for 347 (27.9%), 44 (24.9%), and 383 (29.4%) of preterm infants with the diagnosis of EONS, PNA, and RDS, respectively (Table 2).

**Table 2.** White Blood cell Count Profile in Preterm Infants with EONS, PNA, and RDS.

WBC Count Ranges by Admission Diagnosis			
Ranges (cells/ mm^3^)	EONS (N, %)	PNA (N, %)	RDS (N, %)
	N = 1243	N = 177	N = 1303
<5000	109 (8.8)	16 (9.0)	144 (11.1)
5000-<9100	212 (17.1)	18 (10.2)	164 (12.6)
9100-25 000	575 (46.3)	99 (55.9)	612 (47.0)
>25 000	347 (27.9)	44 (24.9)	383 (29.4)

Abbreviations: WBC = white blood cell count; EONS = early onset newborn sepsis; PNA = perinatal asphyxia; RDS = respiratory distress syndrome.

The HGB value was less than 7 mg/dL in 8 (0.6%) of the preterm infants admitted with a clinical diagnosis of EONS, 3 (1.7%) of preterm infants admitted with a clinical diagnosis of PNA and 5 (0.4%) of preterm infants admitted with a clinical diagnosis of RDS. Two hundred thirty seven (19.1%) preterm infants with EONS were found to be anemic. Polycythemia was found in 81 (6.5%), 8 (4.6%), and 99 (7.6%) of preterm infants with a diagnosis of EONS, PNA, and RDS, respectively (Table 3).

**Table 3.** Hemoglobin Ranges by Admission Diagnosis of Preterm Infants with EONS, PNA and RDS.

Hemoglobin ranges			
Ranges (mg/dL)	EONS (N, %)	PNA (N, %)	RDS (N, %)
	N = 1243	N = 174	N = 1303
<7	8 (0.6)	3 (1.7)	5 (0.4)
7 to <10	16 (1.3)	0	15 (1.2)
10 to <15	213 (17.1)	35 (20.1)	243 (18.6)
15 to 22	925 (74.4)	128 (73.6)	943 (72.3)
>22	81 (6.5)	8 (4.6)	99 (7.6)

Abbreviations: EONS = early onset newborn sepsis; PNA = perinatal asphyxia; RDS = respiratory distress syndrome.

The platelet count was less than 50 000 cells/mm^3^ in 207 (16.8%) of preterm infants admitted with a diagnosis of EONS, in 31 (17.7%) of preterms admitted with a diagnosis of PNA and 254 (19.8%) of preterm infants admitted with a diagnosis of RDS. Platelet counts greater than 450 000 cells/mm^3^ were detected in 12 (1%), 4 (2.3%), and 12 (0.9%) of preterm infants with a diagnosis of EONS, PNA, and RDS, respectively (Table 4).

**Table 4.** Platelet Cell Count Ranges by Admission Diagnosis in Preterm Infants With EONS, PNA, and RDS.

Platelets			
Ranges (cells/ mm^3^)	EONS	PNA	RDS
	N = 1232	N = 175	N = 1282
<50 000	207 (16.8)	31 (17.7)	254 (19.8)
50 000≤100 000	189 (15.3)	31 (17.7)	190 (14.8)
100 000≤150 000	201 (16.3)	25 (14.3)	188 (14.7)
150 000-450 000	623 (50.6)	84 (48.0)	638 (49.8)
≤450 000	12 (1.0)	4 (2.3)	12 (0.9)

Abbreviations: EONS = early onset newborn sepsis; PNA = perinatal asphyxia; RDS = respiratory distress syndrome.

RDS and PNA had statistically significant associations (*P* < .05) with high WBC counts (>25 000 cell/mm^3^) and RDS also had an association with low WBC counts (<5000 cells/mm^3^). EONS did not show a significant association with either low or high WBC counts (Table 5).

**Table 5.** Association Between WBC Abnormalities (<5000 cell/mm^3^ or >25 000 cell/mm^3^) with EONS, PNA, and RDS in Preterm Infants.

Diagnosis	Categories	N	Percent			Normal versus Low			Normal versus High		
			Normal	Low	High	OR	95% CI	*P*-value	OR	95% CI	*P*-value
RDS	Yes	1314	81.66	10.4	7.99	1.302	[1.021, 1.659]	.033	1.616	[1.213, 2.153]	.001
	No	1855	86.36	8.41	5.23	Ref			Ref		
EONS	Yes	1251	84.41	8.71	6.87	0.913	[0.712, 1.172]	.476	1.137	[0.851, 1.518]	.386
	No	1918	84.41	9.54	6.05	Ref			Ref		
PNA	Yes	180	78.89	10.00	11.11	1.172	[0.707, 1.944]	.539	1.96	[1.199, 3.205]	.007
	No	2989	84.74	9.17	6.09	Ref					

Abbreviations: EONS = early onset newborn sepsis; PNA = perinatal asphyxia; RDS = respiratory distress syndrome.

EONS and RDS had significant associations with anemia (HGB < 15 mg/dL), (*P* < .05) while PNA did not have a significant association with anemia (Table 6).

**Table 6.** Association between HGB values of <15 mg/dL with EONS, PNA, and RDS in preterm infants.

Diagnosis	Category	N	% Abnormal	OR	95% CI	*P*-value
RDS	Yes	1316	20.52	1.433	[1.192, 1.722]	.000
	No	1867	15.27			
EONS	Yes	1254	19.38	1.246	[1.035, 1.499]	.020
	No	1929	16.17			
PNA	Yes	177	21.47	1.316	[0.908, 1.907]	.147
	No	3006	17.2			

Abbreviations: EONS = early onset newborn sepsis; PNA = perinatal asphyxia; RDS = respiratory distress syndrome.

Platelet counts of less than 150 000 cell/mm^3^ were significantly associated with EONS and RDS (*P* < .05) but not with PNA (Table 7).

**Table 7.** Association Between Platelet Count Abnormalities of <150 000 cell/mm^3^ or >450 000 cell/mm^3^ with EONS, PNA, and RDS in Preterm infants.

Diagnosis	>Categories	>N	Percent			Normal versus Low			Normal versus High		
			Normal	Low	High	OR	95% CI	*P*-value	OR	95% CI	*P*-value
>RDS	Yes	1293	49.50	49.50	1.01	1.313	[1.138, 0.516]	.000	0.914	[0.459, 1.817]	.798
	No	1846	56.07	42.69	1.25						
>EONS	Yes	1240	50.48	48.55	0.97	1.221	[1.057, 1.411]	.007	0.838	[0.416, 1.687]	.62
	No	1899	55.24	43.5	1.26						
>PNA	Yes	178	47.19	50.56	2.25	1.274	[0.938, 1.730]	.121	2.368	[0.818, 6.849]	.112
	No	2961	53.73	45.19	1.08						

Abbreviations: EONS = early onset newborn sepsis; PNA = perinatal asphyxia; RDS = respiratory distress syndrome.

## Discussion

In this study of blood counts in preterm infants admitted to an NICU in Ethiopia, 1433 (37%) were diagnosed with EONS, 266 (6.9%) were diagnosed with PNA and 1738 (45.3%) were diagnosed with RDS. The mean values for each CBC parameter were 12 490/mm^3^ for WBC counts, 17.9 mg/dL for HGB and 162 992 cells/mm^3^ for platelets.

Leukopenia and leukocytosis are some of the non-specific expected hematologic abnormalities in EONS. Especially when it is severe, the WBC count maybe either below or high (<5000/mm^3^ or >25 000 mm^3^).^13^ Based on this study, about one-third of the preterm neonates were diagnosed with EONS. Nearly 10% of those infants had WBC counts <5000 cells/mm^3^ and one third had a WBC count above 25 000 cells/mm^3^. These percents are substantially lower than in a study done in India, showing leukopenia of <5000 cells/mm^3^ in 25% of neonates with septicemia.^16^

The cut off point for leukocytosis is different in different studies. In our study we used a WBC count of >25 000 cells/mm^3^. Thus, leukocytosis was observed in 27.9% of preterm infants admitted with a clinical diagnosis of EONS which is comparable with a study done in India by Panwar et al in both term and preterm infants that showed a leukocytosis rate of 30.5% for blood culture positive infants and a leukocytosis rate of 20.4% for blood culture negative infants. However, these numbers are different from the reported ranges (1.3%-17%) published elsewhere.^10^ Based on our study, EONS does not show a statistically significant association with WBC count abnormalities.

Thrombocytopenia is one of the most common hematological abnormalities in neonates, affecting about 22% to 35% of NICU admissions. Eight percent of admitted preterm babies are reported to develop severe thrombocytopenia (<50 000 cells/mm^3^) because of many reasons.^17,18^ In the present study, lower platelet counts (<150 000 cells/mm^3^) were found in 48.5% of preterm infants with EONS, of which 16.8% had platelet counts less than 50 000 cells/mm^3^. The prevalence of thrombocytopenia in preterm infants with EONS in our study is higher than in a study done in Pakistan and India both in term and preterm babies (thrombocytopenia rates of 24.7% and 28.7%, respectively) but lower than that of another study done in India (thrombocytopenia rate of 83.5%).^18,19^

In this study, thrombocytopenia was found in 49.3% and 48.5% of preterm infants with RDS and EONS respectively which is comparable to studies done in Bellevue Hospital, USA and in India where thrombocytopenia was detected in 42% and 31.6% of babies with RDS and sepsis, respectively^20^ but lower than a study in Pakistan that showed a low platelet count in 75.6% of newborns.^21^ Our study showed a significant association between a low platelet count and EONS, PNA, and RDS with *P*-values of <.05.

Sepsis can cause anemia through bone marrow dysfunction or bleeding (petechiae, purpura, oozing) and hemolysis.^22^ In this study, HGB levels less than 15 mg/dL (anemia) were observed in 20.2% of neonates and had a significant association with EONS as well as RDS (*P* < .05), but the association with PNA was not significant. Changes in the hematopoietic system can be observed as complications of asphyxia. Changes in the structure and function of erythrocytes, leukocytes, and thrombocytes can be caused by asphyxia.

Less than 1% of babies in a well-baby nursery are expected to have a platelet count <150 000/mm.^3,23^ However, in NICUs, thrombocytopenia is common with a reported incidence of 18% to 35%. In this study, 7.2% of the study subjects were diagnosed with PNA. Thrombocytopenia (<150 000/mm^3^) was detected in 49.7% of preterm newborns with PNA of which 17.7% had platelet count <50 000 cells/mm^3^, 17.7% between 50 000 cells/mm^3^ and 100 000 cells/mm^3^ and 15.6% had a platelet count of between 100 000 to 150 000 cells/mm^3^. This finding is not consistent with similar studies from USA and India, which detected thrombocytopenia in 31% of preterm newborns with PNA.^11^ This disparity is believed to reflect the severe form of PNA that many newborns in developing countries sustain. In another study from West Bengal, India, thrombocytopenia was detected in 24% of newborns with PNA.

About 73.6% of preterm newborns with PNA in our study had a hemoglobin value of 15 to 22 g/dL. Severe anemia with a HGB value of <7 g/dL was found in 1.7% of asphyxiated preterm newborns but it did not have a statistically significant association with PNA. Polycythemia, with a defined HGB value of >22 mg/dL, was detected in 4.60% of preterm newborns with PNA. This agrees with findings from India, where the mean HGB value for asphyxiated newborns was found to be 16.5 g/dL ± 2.57.^24^ In a study from Slovakia on asphyxiated newborns, the mean HGB value of asphyxiated newborns was 16.66 ± 5.4 g/dL.^25^

In our study, among preterm newborns with PNA, 9.0% had a total WBC count of <5000/mm^3^ and 24.9% had WBC counts in the range of >25 000 cells/mm^3^ which had a significant association with asphyxia. The WBC count was 9100 to 25 000/mm^3^ in the majority of asphyxiated preterm newborns (55.9%). The mean WBC count for preterm newborns was 5700 in a Japanese cohort study.^26^

Although we could not find a study done on hematologic profile of preterm neonates with acute RDS, the oxygen content of the blood depends on the HGB level, cardiac output and lung ventilation. Neonates with RDS commonly have low oxygenation levels because of insufficient (or dysfunctional) surfactant, where they develop generalized atelectasis, ventilation-perfusion mismatching, with subsequent hypoxemia and respiratory acidosis.^8^ In this study, the HGB level was less than 15 mg/dL for 19.9% of babies with a clinical diagnosis of RDS.

Based on this study, the platelet count of those neonates with RDS was less than 50 000 cells/mm^3^ and between 50 000 and 100 000 cells/mm^3^ for 19.8% and 14.8% of babies, respectively.

The WBC count of the babies with RDS was less than 5000 cells/mm^3^ for 11.1% and above 25 000 cells/mm^3^ for 29.4%. In this study, both low and high WBC counts, anemia and thrombocytopenia were significantly associated with RDS, all having *P*-values of <.05.

An important limitation of our study is that the machines used to determine the CBC were different in each hospital. The hematologic abnormality in this study may be affected by other problems of prematurity when multiple conditions were present at the same time. Another limitation of our study is that we did not include the mean platelet volume (MPV), neutrophil-to-lymphocyte ratio (NLR), platelet-to-lymphocyte ratio (PLR), and MPV/platelet ratios.

In conclusion, the hematologic profile of preterm infants is affected by RDS, EONS, and PNA in low-resource countries such as Ethiopia. Several authors have reported hematologic data for term neonates. However, these data may not apply to preterm newborns whose blood parameters vary widely from one individual to another. The fact that many preterm newborns have medical complications related to their gestational age that will affect blood parameters, suggests that these newborns need separate standards for hematologic profiling. We, therefore; recommend further study on the effect of the hematologic abnormalities on the subsequent outcome of preterm babies.
